# 878. Clinical impact of plasma cell-free metagenomic next-generation sequencing: A retrospective cohort study 

**DOI:** 10.1093/ofid/ofad500.923

**Published:** 2023-11-27

**Authors:** Shilpa Vasishta, Christopher Vo, Sushrita Neogi, Bayan Alahmdi, Timothy Sullivan, Melissa R Gitman

**Affiliations:** Montefiore Medical Center, New York, NY; Icahn School of Medicine at Mount Sinai, NEW YORK, New York; Icahn School of Medicine at Mount Sinai, NEW YORK, New York; Icahn School of Medicine at Mount Sinai, NEW YORK, New York; Icahn School of Medicine at Mount Sinai, NEW YORK, New York; Icahn School of Medicine at Mount Sinai, NEW YORK, New York

## Abstract

**Background:**

Plasma cell-free metagenomic next-generation sequencing (cf-mNGS) offers potential for rapid, noninvasive diagnosis of complex infections, though clinical impact remains incompletely characterized. Here, we describe impacts on diagnosis and antimicrobial management among patients evaluated with plasma cf-mNGS.

**Methods:**

Patients evaluated with plasma cf-mNGS (Karius®) in the Mount Sinai Health System from January 1, 2019 to December 23, 2022 were included. Cases were each reviewed independently in the medical record by two investigators using a standardized data collection form with discrepancies adjudicated collectively. Data were analyzed descriptively.

**Results:**

Plasma cf-mNGS was sent for 37 patients of whom 29 (78%) were immunocompromised. 26 (70%) had prior invasive diagnostics. Median time from admission to NGS was 15 days. NGS was positive in 24 cases (65%), with results reflecting 20 bacterial infections, 9 viral, and 4 fungal (14 monomicrobial, 10 polymicrobial). Results were deemed clinically relevant in 18 cases (49%) including 12 positive and 6 negative tests. Testing led to antimicrobial changes in 11 cases (30%), discontinuation in 3 cases (8%), and no change in 23 cases (62%). After plasma cf-mNGS (compared with prior), fewer patients received 3 or 4 antimicrobials, while more received 0, 1, or 2 antimicrobials (Fig. 1A); fewer patients received combined antibiotic/antifungal therapy, while more received no antimicrobials or antifungal therapy alone; there was no change in use of antibiotic therapy alone (Fig. 1B). Final diagnoses included 15 bacterial infections (41%), 9 fungal (24%), 0 viral, and 7 noninfectious conditions (19%). Final diagnoses were attributed to NGS in 6 cases (16%), other diagnostics in 9 cases (24%), and both NGS and other diagnostics in 6 cases (16%); in the latter group, NGS provided a diagnosis prior to another study in 3 cases by intervals of 13, 19, and 72 days, respectively.

**Figure:**
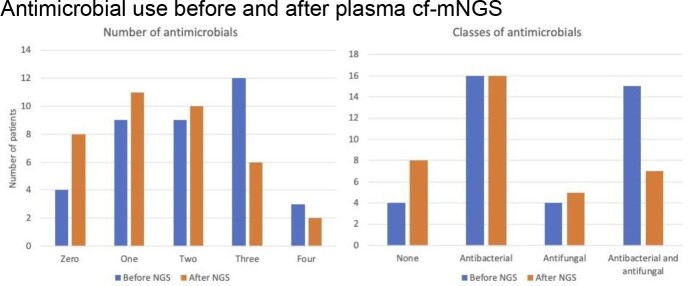

A. Changes in number of antimicrobials, B. Changes in classes of antimicrobials

**Conclusion:**

Plasma cf-mNGS, as an adjunct to traditional methods, may provide unique or expedited diagnosis of bacterial or fungal infections, accelerate diagnosis of noninfectious conditions, or inform changes in broad-spectrum antimicrobial regimens. Continued study is warranted to delineate clinical impact and optimal use of plasma cf-mNGS.

**Disclosures:**

**All Authors**: No reported disclosures

